# Elective neck dissection improves the survival of patients with T2N0M0 oral squamous cell carcinoma: a study of the SEER database

**DOI:** 10.1186/s12885-021-09053-3

**Published:** 2021-12-07

**Authors:** Alimujiang Wushou, Feiluore Yibulayin, Lu Sheng, Yuan Luo, Zhi-cheng Yang

**Affiliations:** 1 Department of Oral & Maxillofacial Surgery, Shanghai Key Laboratory of Craniomaxillofacial Development and Diseases Shanghai Stomatological Hospital,, Fudan University, 356 Beijing East Road, Shanghai, 200001 PR China; 2 Shanghai Key Laboratory of Craniomaxillofacial Development and Diseases, Fudan University, 356 Beijing East Road, Shanghai, 200001 PR China; 3 Department of Preventive Medicine, School of Public Health, Shanghai Medical College, Fudan University, Shanghai, China

**Keywords:** Oral squamous cell carcinoma, T2N0M0, Elective neck dissection, Surgical treatment, SEER database

## Abstract

**Background:**

Treatment of clinical N0 neck tumours is controversial in early-stage oral squamous cell carcinoma (OSCC), possibly because T1N0M0 and T2N0M0 merge together at early stages. The purposes of this study were to compare survival outcomes only for T2N0M0 cases based upon treatment elective neck dissection versus neck observation.

**Methods:**

T2N0M0 OSCC cases were identified in the Surveillance, Epidemiology, and End Results database of the United States National Cancer Institute between 2004 and 2015. Survival curves for different variable values were generated using Kaplan-Meier estimates and compared using the log-rank test. Variables that achieved significance at *P* < 0.05 were entered into multivariable analyses via the Cox proportional hazards multivariate regression.

**Results:**

A total of 2857 patients were selected, and 2313 cases were available for disease specific survival (DSS). The 5-year and 10-year overall survival (OS) were 66.7 and 46% for patients receiving elective neck dissection (END), respectively, and 56.4 and 37.2% for patients with neck observation (*P <* 0.0001). The 5-year and 10-year DSS were 73.6 and 64% for the END group, respectively, versus 64.5 and 54.5% for the neck observation group (*P <* 0.0001). More importantly, performing END was independently associated with favourable DSS and OS for patients with T2N0M0 OSCC [hazard ratio (HR) = 0.769, *P* = 0.0069 for DSS; HR = 0.829, *P* = 0.0031 for OS, neck observation group as reference] according to multivariate survival analysis.

**Conclusion:**

END is recommended for T2N0M0 OSCC cases and it is associated with improved DSS and OS.

## Background

Head and neck squamous cell carcinoma is increasingly prevalent worldwide and is the 16th leading cause of cancer death. Oral squamous cell carcinoma (OSCC) is the most common cancer of the oral cavity, representing more than 95% of all cases and making up almost half of newly diagnosed head and neck squamous cell carcinoma [1, 2]. Overall survival (OS) rates of OSCC varied from 10 to 82% depending on various prognostic factors, including age, race, sex, primary site, drinking, smoking, human papillomavirus status, American Joint Committee on Cancer (AJCC) stage, pathological differentiation and treatment modalities [3]. Among these prognostic factors distant metastasis is one of the strongest indicators. Another important prognostic factor in survival is the presence of lymph node metastasis. It has been reported that cervical lymph node metastasis and extranodal extension are prerequisites for distant metastasis development [4].

The existence of cervical lymph node metastasis demonstrates the most important clinico-pathological prognostic factor. The presence of even one positive cervical lymph node is associated with a 50% reduction in the OS [5]. Thus, appropriate treatment of the cervical lymph node is as important as treating the primary site to achieve good oncologic results. The optimal treatment protocol for patients with early stage (T1–2N0M0) tumours has been debated in the past several decades; no consensus has been reached because of similar prognosis between elective neck dissection (END) and neck observation. Head and neck surgical oncologists prefer preventive END to avoid regional recurrence; supraomohyoid neck dissection is well established [6]. However, others believe END is an aggressive regime, especially for young female patients with T1N0M0 tumours, mostly because of the neck contour changes and some surgical complications. Therefore, a wait-and-watch policy is recommended, which favours regular consultation without simultaneous neck dissection [7].

The AJCC T stage is an independent prognostic indicator, and T2-stage OSCC has demonstrated worse prognosis than T1 [8]. However, most previous studies have merged T1 and T2 in early stages of evaluating prognosis [9]. When clinically dealing with T1- and T2-stage OSCC, surgeons have encountered challenging treatment strategies. Surgically treated T1-stage OSCC typically does not require defect repair or postoperative defects can be closed with adjacent flaps. However, the postoperative defect of T2-stage tumours often requires free flap repair and simultaneous END- facilitated oral defect reconstruction, which would improve the patient quality of life [10]. Therefore, when assessing prognosis, T1N0M0 and T2N0M0 stage tumours should be separately evaluated instead of together [11]. In view of organ preservation or surgical reconstruction, we believe that surgical treatment is better than radiotherapy, chemotherapy or their combination for T2N0M0 OSCC patients. Furthermore, simultaneously performing END can prevent regional recurrence and is helpful for performing free flap reconstruction. Lastly, postoperative defect repair and functional restoration will eventually improve overall survival. Here, in order verify our above postulated conditions, we present a retrospective investigation comparing survival outcomes only for T2N0M0 cases based upon treatment END versus neck observation.

## Methods

### Study cohort

The study population was extracted from the Surveillance, Epidemiology, and End Results (SEER) database of the United States National Cancer Institute using its software (https://seer.cancer.gov, SEER*Stat 8.3.6). Patients were identified via the International Classification of Diseases for Oncology, Third Edition (ICD-O-3) as previously described [12]. Briefly, the OSCC cases were selected through the ICD-O-3 morphologic and topographic codes: 8050–8076, 8078, 8083, 8084, 8094, C01.9, C02.0, C02.1, C02.2, C02.8, C02.9, C03.0, C03.1, C03.9, C04.0, C04.9, C05.0, C06.0, C06.1 and C06.2.

We selected only pathologically confirmed T2N0M0 (AJCC stage II) OSCC primary cases. Patients receiving neck dissection were identified through the SEER fields for regional lymph node surgery. The variables in the analysis included tumour origination, marital status at diagnosis, age at diagnosis, sex, race, pathological differentiation, whether neck dissection was performed, treatment modalities, vital status and follow-up period. Our study used the established data and did not involve interactions with human subjects. Therefore, institutional review board approval was not required.

### Statistical analysis

Differences in numerical variables were evaluated with Student’s test or the non-parametric Wilcoxon test. Categorical variables were compared by the chi square test or Fisher exact test. Survival analysis were performed using the Kaplan-Meier estimates. Independent prognostic factors were identified via the Cox proportional hazards multivariate regression. Data analyzation were carried out applying Statistical Package for Social Sciences, Version 23.0, for Windows (SPSS, Chicago, IL) and statistical packages R (The R foundation; http://www.r-project.org; version 3.4.3), Empower R (http://www.empowerstats.com, Boston, MA).

## Results

### Clinicopathologic characteristics

A total of 2857 patients were selected. The cohort consisted of 1691 males and 1166 females with a mean age of 64 years. Caucasian accounted for 84.6% (2418/2857) and black American 6% (172/2857) of the study population. The overall mean follow-up period was 54.2 months (range, 0–155 months). In more than half of the cases (1611/2857, 56.4%), the orientation was tongue. END was performed for 62.5% (1787/2857) patients. The overall clinicopathologic characteristics are summarized in Table 1.

**Table 1:** Clinico-pathological characteristic of study population

Parameters		Overall survival						Disease specific survival					
		Neck observation			Neck dissection			Neck observation			Neck dissection		
		Alive	Dead	*P*-value	Alive	Dead	*P*-value	Alive	Dead	*P*-value	Alive	Dead	*P*-value
**Tumor origination**	Floor of mouth	63	75	0.396	176	130	0.000	62	29	0.678	172	77	0.000
	Gum and Other Mouth	179	179		249	195		177	96		244	111	
	Tongue	298	275		696	340		291	168		689	195	
**Marital status at diagnosis**	Single	76	83	0.000	196	113	0.000	76	43	0.000	191	72	0.441
	Married	330	220		646	336		323	136		638	216	
	Other status	111	198		217	192		108	99		214	87	
**Age period**	20–29	2	2	0.000	17	4	0.000	2	2	0.000	17	3	0.000
	30–39	18	3		65	15		18	2		65	15	
	40–49	54	28		179	58		53	23		177	44	
	50–59	137	72		325	137		137	49		320	92	
	60–69	139	119		296	194		138	64		292	104	
	70–79	110	136		182	163		105	77		177	79	
	80+	80	169		57	94		77	76		57	46	
**Age at diagnosis**	Age ≤ 64	294	159	0.000	763	306	0.000	292	102	0.000	755	201	0.000
	Age > 64	246	370		358	359		238	191		350	182	
**Gender**	Female	249	237	0.667	427	251	0.884	242	137	0.265	419	139	0.899
	Male	291	292		694	414		288	156		686	224	
**Race**	White	464	462	0.653	920	570	0.100	454	251	0.786	908	322	0.623
	Black	25	29		76	42		25	17		74	26	
	Others	46	37		119	51		46	24		117	34	
**Pathological grade**	Grade I	177	133	0.006	242	122	0.013	175	55	0.000	237	59	0.000
	Grade II	236	263		683	398		232	154		674	229	
	Grade III + IV	73	93		161	129		71	66		160	87	
**Radiotherapy**	No	345	324	0.372	694	378	0.035	339	159	0.006	685	191	0.000
	Yes	195	205		427	287		191	134		420	192	
**Chemotherapy**	No	478	471	0.789	1034	599	0.076	469	250	0.022	1020	331	0.001
	Yes	62	58		84	66		61	43		85	52	
**Treatment**	Surgery	345	323	0.478	694	378	0.075	339	158	0.022	685	191	0.000
	Surgery +RT	133	147		340	221		130	91		335	140	
	Surgery + RT + chemotherapy	62	57		87	66		61	43		85	52	

For disease specific survival (DSS) analysis, 2313 cases were available, including 939 females and 1374 males. The mean follow-up period for DSS was nearly the same as OS. END was performed in 1488 cases in this cohort. Summary statistics of DSS status are presented in Table 1. When stratified by histologic grade, 528 cases were of grade I, 1289 cases of grade II, and the remaining 384 cases were of grades III and IV. Histologic grade information was missing for 111 cases. According to the distribution of sample sites, we divided the study population into three groups: (A) floor of mouth, (B) tongue and (C) other sites combined, including upper gum, lower gum, hard palate, cheek mucosa, vestibule of mouth and retromolar area.

#### Survival analysis

**Fig. 1:**
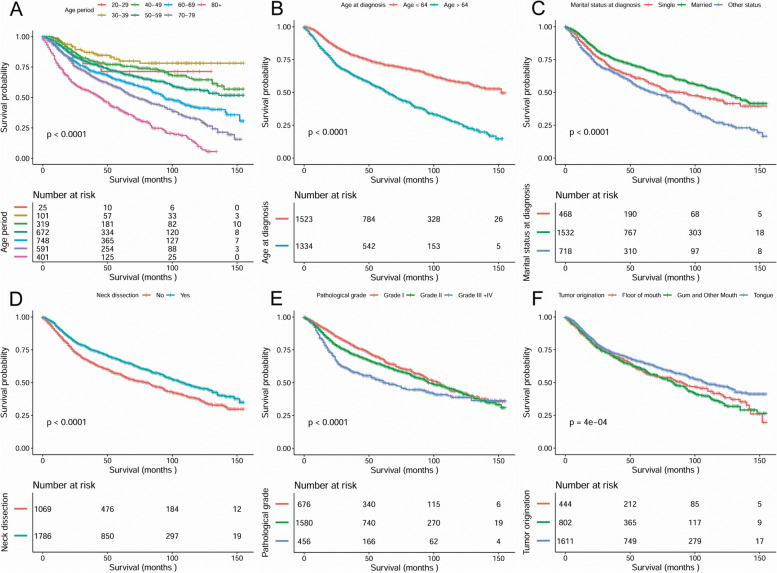
Overall survival curves of cases with T2N0M0 OSCC compared according to (**A**) age range, (**B**) mean age at diagnosis, (C) marital status at diagnosis, (**D**) neck dissection, (**E**) pathological differentiation, (**F**) tumor orientation

Kaplan-Meier analysis was applied for time-to-event analysis. Regardless of all other factors, the 5-year and 10-year OS were 66.7 and 46%, respectively, for patients receiving END and 56.4 and 37.2% for patients with neck observation (*P* < 0.0001, Fig. 1). Significant OS differences were found depending on age range (*P <* 0.0001), mean age (*P <* 0.0001), marital status at diagnosis (*P <* 0.0001), pathological grade (*P <* 0.0001) and tumour orientation (*P <* 0.0001) (Fig. 1).

**Fig. 2:**
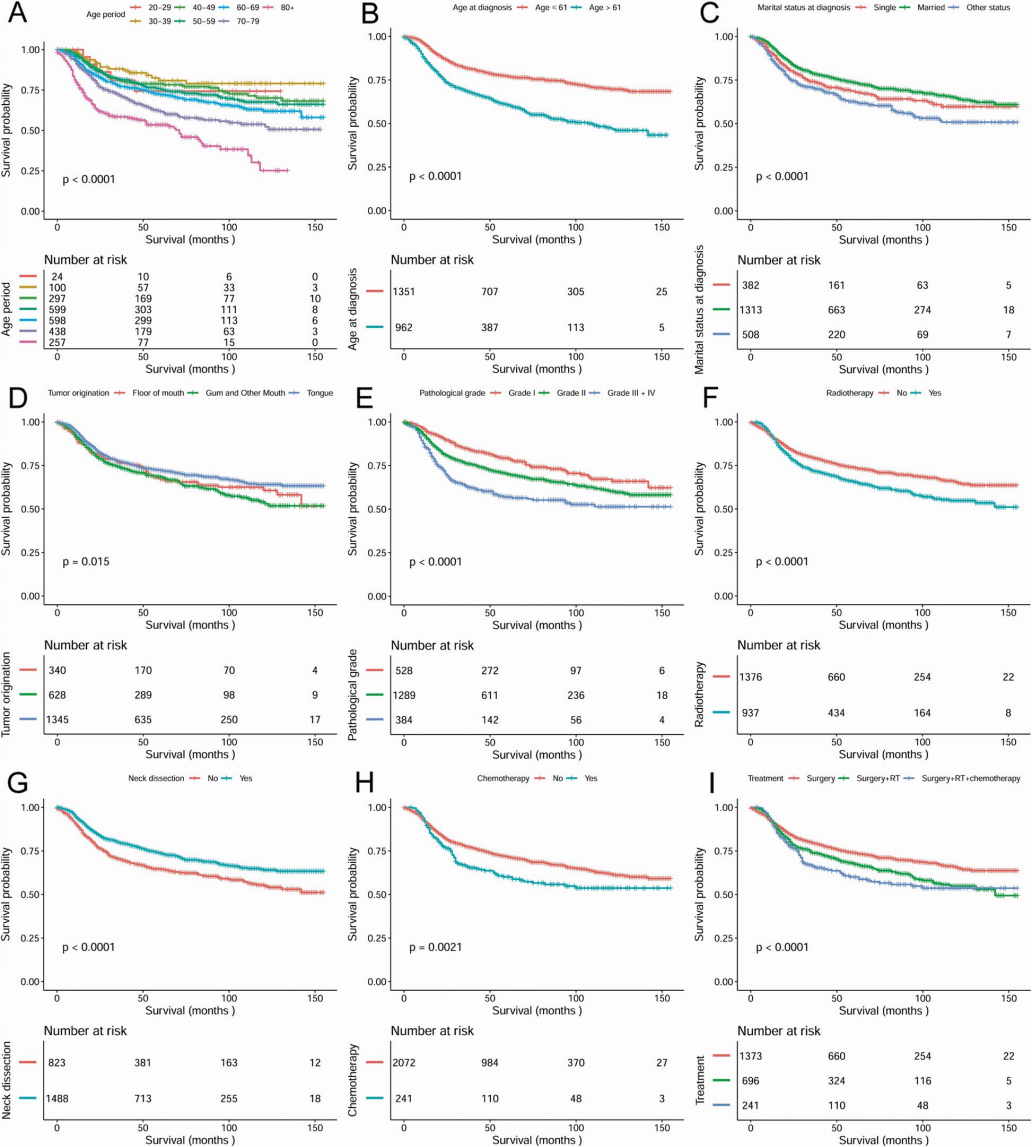
Disease specific survival curves of cases with T2N0M0 OSCC compared according to (**A**) age range, (**B**) mean age at diagnosis, (**C**) marital status at diagnosis, (**D**) tumor orientation, (**E**) pathological differentiation, (**F**) radiotherapy, (**G**) neck dissection, (**H**) chemotherapy and (**I**) treatment modalities

In the survival analysis for DSS, patients treated with END showed significantly higher survival rates than patients in the neck observation group (*P <* 0.0001). The 5-year and 10-year DSS for all causes combined were 73.6 and 64% for the END group, respectively, versus 64.5 and 54.5% for the neck observation group. Additionally, remarkable survival differences were identified depending on age range (*P <* 0.0001), mean age (*P <* 0.0001), marital status at diagnosis (*P* < 0.0001), tumour orientation (*P* = 0.015), pathological grade (*P <* 0.0001), radiotherapy (*P <* 0.0001), chemotherapy (*P* = 0.0021) and different treatment modalities (*P <* 0.0001) (Fig. 2).

#### Cox regression multivariate analysis

To determine the efficacy of END based on various clinicopathological factors, all significant factors in the survival analysis were entered into the multivariable analysis based on the Cox regression model.

The END was favourably associated with better DSS and OS [hazard ratio (HR) 95% confidence interval (CI) = 0.769 (0.675–0.939), *P* = 0.0069 for DSS; HR (95% CI) = 0.829 (0.732–0.939), *P* = 0.0031 for OS, neck observation as reference].

**Fig. 3:**
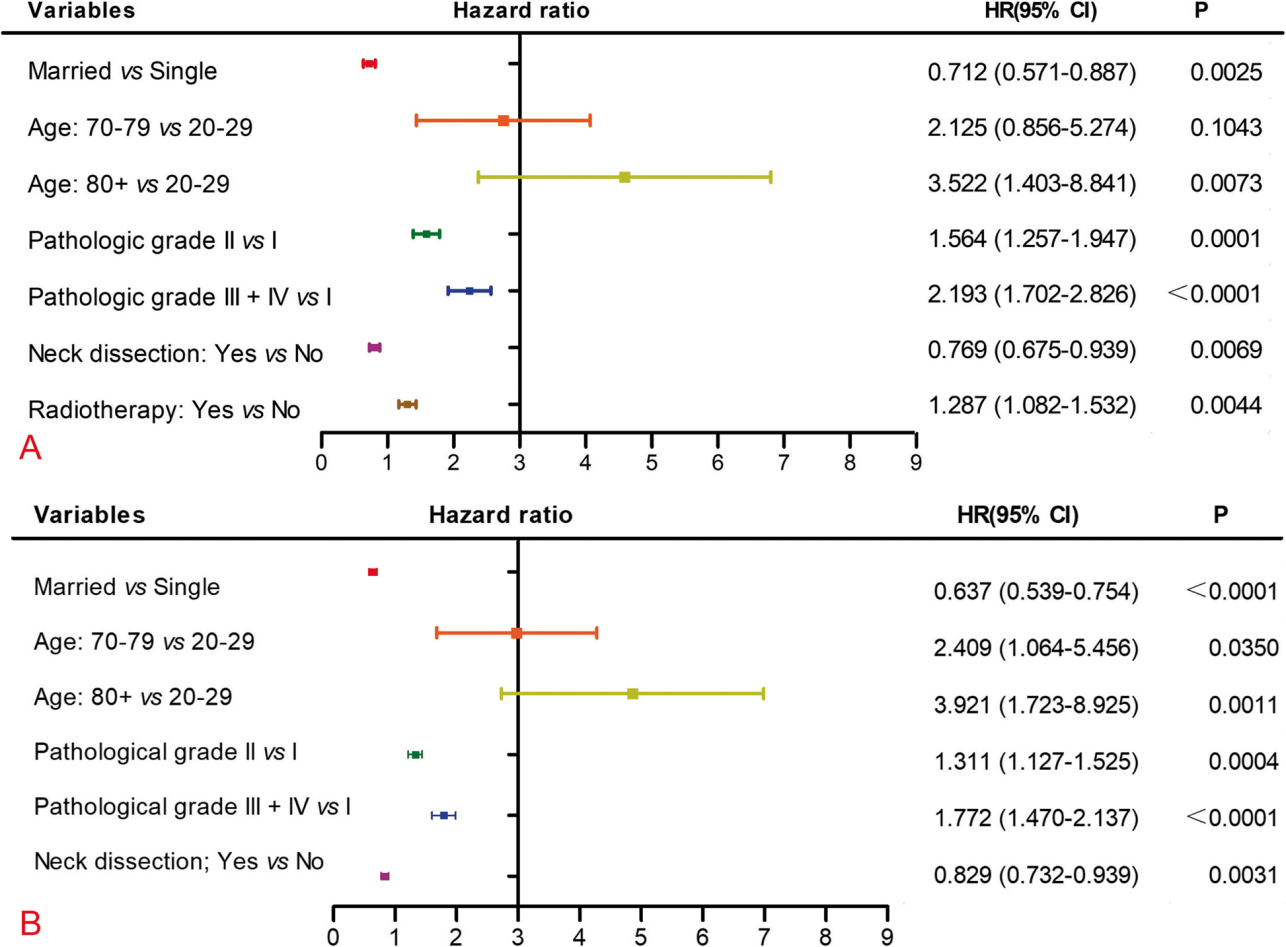
Forest plots summarizing hazard ratios for (**A**) DSS and (**B**) OS

The higher pathological grades were adversely associated with DSS and OS [Grade II HR (95% CI) = 1.564 (1.257–1.947), *P* = 0.0001; Grade III + IV, HR (95% CI) = 2.193 (1.702–2.826), *P <* 0.0001 for DSS; Grade II, HR (95% CI) = 1.311 (1.127–1.525), *P* = 0.0004; Grade III + IV, HR (95% CI) = 1.772 (1.47–2.137), *P <* 0.0001 for OS, Grade I as reference]. The details of the multivariate Cox regression analysis are presented in Fig. 3.

## Discussion

Management of the clinical N0 neck tumour is controversial in early OSCC [13]. The metastasis rate of the cervical lymph node is inconsistent according to previous reports. Most importantly, occult metastases exist in early stage OSCC [14]. Previous studies have demonstrated that the occult metastasis rate was approximately 25% in for OSCC patients treated with early-stage END [15–18]. It may be concluded that one in four patients is exposed to the risk of regional recurrence or even developing distant metastasis. However, few studies have clarified whether these patients were T1N0M0 or T2N0M0 and further did not evaluate the effects of performing END on prognosis. This report, to our knowledge, analyses the largest population of T2N0M0 OSCC patients independently to date. We found that performing END effectively improved T2N0M0 OSCC survival and is an independent prognostic indicator.

Based on our results, pathological differentiation is another important prognostic indicator. Compared with grade I well-differentiated OSCC cases, the other three grades (Grades II, III and IV) are adversely associated with DSS and OS. The tumour subtype is an important prognostic factor that has been neglected in previous studies in deciding whether to use END. There are many variants of OSCC, having different biological behaviours and prognosis [19–21]. Almost all SCC variants are registered in the SEER database. To ensure the accuracy of our results, we filtered two variants (8070/3 and 8071/3) and found no significant survival differences between the two variants (*P* >0.05).

Regarding oral solid neoplasm, surgical resection remains the most effect treatment modality [22]. Except for small T1-stage tumour postoperative defects, OSCC surgical treatment often involves resection and functional reconstruction [4]. Surgery is irreplaceable for T2N0M0 OSCC patients. Radiotherapy, chemotherapy or their combination cannot achieve as good results as surgery in oral function recovery. We also studied cases treated with methods other than surgery and evaluated their prognosis. The results demonstrated that there were significant survival differences among the various treatment modalities. Patients treated with surgery showed better prognosis than those treated with other means alone or combinations. Without a doubt, the adjuvant roles of radiotherapy and chemotherapy should be admitted in the positive margin of pathologically undifferentiated cases.

There are variances in the survival rates of different tumour orientations [4]. Controversy sexist in the management of T2N0M0 OSCC arising from maxillary gingiva, alveolus, and hard palate [23]. The low incidence of cervical metastases has historically been considered a hallmark of this disease, and a “watch-and-wait” strategy is typically used to control neck lymph node metastases [24, 25]. The results of survival analysis according to the OSCC orientation showed that group A (floor of mouth) and B (tongue) demonstrated better DSS and OS prognosis than group C (other sites). Based on this finding, we concluded that T2N0M0 OSCC of maxillary gingiva, alveolus, and hard palate should be considered as equally aggressive as those at other sites. However, this conclusion may require further subgroup confirmation.

A few limitations of the publicly available SEER database and the current investigation should be acknowledged. First, not all cases have complete information, including important variables such as pathological grade, HPV status and detailed chemotherapy. Second, due to the lack of oral defect reconstruction data, the role of free flap construction for life quality improvement could not be well established. Third, the follow-up period was variable (0–155 months). Finally, the retrospective nature of the current study may have introduced bias into the overall analysis.

## Conclusion

Most previous retrospective or prospective T1/2N0M0 OSCC studies regarding END had small sample sizes. Decisions on whether to proceed with END versus a “wait-and-watch” approach in T1/2N0M0 is controversial for the following reasons. First, the disease has relatively low incidence. Second, early-stage tumours require much longer follow-up durations to identify differences between the two treatment strategies. Finally, and most importantly, radiochemotherapy has shown similar survival rates compared with surgery in early stage OSCC. Despite the limitation of incomplete data and the study itself, the present investigation is the first of its kind using the largest study population from multiple orientations to show that patients with T2N0M0 OSCC benefited from END associating with improved DSS and OS and it was an important independent prognostic factor. Thus, performing END is recommended for patients with T2N0M0 OSCC cases.

## Data Availability

Study data was publicly available in the SEER database (https://seer.cancer.gov).

## Abbreviations

OSCC: Squamous cell carcinoma
OS: Overall survival
AJCC: American Joint Committee on Cancer
END: Elective neck dissection
SEER: Surveillance, Epidemiology, and End Results
ICD-O-3: International Classification of Diseases for Oncology, Third Edition
SPSS: Statistical Package for Social Sciences
DSS: Disease specific survival
HR: Hazard ratio
CI: Confidence interval

## References

[1] Siegel RL, Miller KD, Jemal A (2019). Cancer statistics, 2019. CA Cancer J Clin.

[2] Chaturvedi AK, Anderson WF, Lortet-Tieulent J, Curado MP, Ferlay J, Franceschi S, Rosenberg PS, Bray F, Gillison ML (2013). Worldwide trends in incidence rates for oral cavity and oropharyngeal cancers. J Clin Oncol.

[3] Bray F, Ferlay J, Soerjomataram I, Siegel RL (2018). Global cancer statistics 2018: GLOBOCAN estimates of incidence and mortality worldwide for 36 cancers in 185 countries. CA Cancer J Clin.

[4] Chinn SB, Myers JN (2015). Oral cavity carcinoma. Current management, controversies, and future directions. J Clin Oncol.

[5] Cerezo L, Millán I, Torre A, Aragón G, Otero J (1992). Prognostic factors for survival and tumor control in cervical lymph node metastases from head and neck cancer. A multivariate study of 492 cases. Cancer.

[6] Kelner N, Vartanian JG, Pinto CA, Coutinho-Camillo CM, Kowalski LP (2014). Does elective neck dissection in T1/T2 carcinoma of the oral tongue and floor of the mouth influence recurrence and survival rates?. Br J Oral Maxillofac Surg.

[7] Cao Y, Wang T, Yu C, Guo X, Li C, Li L (2019). Elective neck dissection versus wait-and-watch policy for Oral cavity squamous cell carcinoma in early stage: a systematic review and Meta-analysis based on survival data. J Oral Maxillofac Surg.

[8] Ebrahimi A, Gil Z, Amit M, Yen T-C, Liao C-T, Chaturvedi P, Agarwal JP, Kowalski LP, Kreppel M, Cernea CR (2014). Primary tumor staging for oral cancer and a proposed modification incorporating depth of invasion: an international multicenter retrospective study. JAMA Otolaryngol Head Neck Surg.

[9] Cai H, Zhu Y, Wang C, Zhang Y, Hou J (2020). Neck nodal recurrence and survival of clinical T1-2 N0 oral squamous cell carcinoma in comparison of elective neck dissection versus observation: a meta-analysis. Oral Surg Oral Med Oral Pathol Oral Radiol.

[10] Feng Z, Li JN, Li CZ, Guo CB (2014). Elective neck dissection versus observation in the management of early tongue carcinoma with clinically node-negative neck: a retrospective study of 229 cases. J Craniomaxillofac Surg.

[11] Feng Z, Cheng A, Alzahrani S, Li B, Han Z, Ward BB (2020). Elective neck dissection in T1N0M0 Oral squamous cell carcinoma: when is it necessary?. J Oral Maxillofac Surg.

[12] Fakhry C, Krapcho M, Eisele DW, D'Souza G. Head and neck squamous cell cancers in the United States are rare and the risk now is higher among white individuals compared with black individuals. Cancer. 2018;124(10):2125–33. 10.1002/cncr.31322 PMC5953421 29533459

[13] Massey C, Dharmarajan A, Bannuru RR, Rebeiz E (2019). Management of N0 neck in early oral squamous cell carcinoma: a systematic review and meta-analysis. Laryngoscope.

[14] Zhangfan D, Tingying X, Jie H, Yihang Y, Qingsong Y (2019). Elective neck dissection versus observation in squamous cell carcinoma of Oral cavity with clinically N0 neck: a systematic review and Meta-analysis of prospective studies. J Oral Maxillofac Surg.

[15] Huang SF, Kang C-J, Lin C-Y, Fan K-H, Yen T-C, Wang H-M, Chen I-H, Liao C-T, Cheng A-J, Chang JT-C (2008). Neck treatment of patients with early stage oral tongue cancer: comparison between observation, supraomohyoid dissection, and extended dissection. Cancer.

[16] Keski-Santti H, Atula T, Tornwall J, Koivunen P, Makitie A (2006). Elective neck treatment versus observation in patients with T1/T2 N0 squamous cell carcinoma of oral tongue. Oral Oncol.

[17] Ho CM, Lam KH, Wei WI, Lau SK, Lam LK (1992). Occult lymph node metastasis in small oral tongue cancers. Head Neck.

[18] Li Y, Liu K, Ke Y, Zeng Y, Chen M, Li W, Liu W, Hua X, Li Z, Zhong Y (1992). Risk factors analysis of pathologically confirmed cervical lymph nodes metastasis in Oral squamous cell carcinoma patients with clinically negative cervical lymph node: results from a Cancer Center of Central China. Head Neck.

[19] Fritsch VA, Lentsch EJ (2014). Basaloid squamous cell carcinoma of the head and neck: location means everything. J Surg Oncol.

[20] Alonso JE, Kuan EC, Arshi A, St John MA (2018). A population-based analysis of verrucous carcinoma of the oral cavity. Laryngoscope.

[21] Bice TC, Tran V, Merkley MA, Newlands SD, van der Sloot PG, Wu S, Miller MC (2015). Disease-specific survival with spindle cell carcinoma of the head and neck. Otolaryngol Head Neck Surg.

[22] Rogers SN, Brown JS, Woolgar JA, Lowe D, Magennis P, Shaw RJ, Sutton D, Errington D, Vaughan D (2009). Survival following primary surgery for oral cancer. Oral Oncol.

[23] Cariati P, Cabello-Serrano A, Monsalve-Iglesias F, Fernadez-Solis J, Martinez-Lara I (2019). Is a “watch and wait strategy” safe to manage clinically N0 squamous cell carcinoma of the upper jaw?. Curr Probl Cancer.

[24] de Bree R, Takes RP, Shah JP, Hamoir M, Kowalski LP, Robbins KT, Rodrigo JP, Sanabria A, Medina JE, Rinaldo A (2019). Elective neck dissection in oral squamous cell carcinoma: past, present and future. Oral Oncol.

[25] Abu-Ghanem S, Yehuda M, Carmel NN, Leshno M, Abergel A, Gutfeld O, Fliss DM (2016). Elective neck dissection vs observation in early-stage squamous cell carcinoma of the oral tongue with no clinically apparent lymph node metastasis in the neck: a systematic review and Meta-analysis. JAMA Otolaryngol Head Neck Surg..

